# Are the ways women cope with stressors related to their health behaviors over time?

**DOI:** 10.1093/abm/kaaf006

**Published:** 2025-02-06

**Authors:** Claudia Trudel-Fitzgerald, Scott G Smith, Laura D Kubzansky

**Affiliations:** Department of Psychology, Université du Québec à Trois-Rivières, Trois-Rivières, Québec, G8Z 4M3, Canada; Research Center, Institut Universitaire en Santé Mentale de Montréal, Montréal, Québec, H1N 3V2, Canada; Lee Kum Sheung Center for Health and Happiness, Harvard T.H. Chan School of Public Health, Boston, Massachusetts, 02115, United States; Department of Epidemiology, Harvard T.H. Chan School of Public Health, Boston, Massachusetts, 02115, United States; Department of Social and Behavioral Sciences, Harvard T.H. Chan School of Public Health, Boston, Massachusetts, 02115, United States

**Keywords:** coping skills, coping variability, emotion regulation, health behavior, lifestyle

## Abstract

**Objective:**

Emerging research suggests the use of certain strategies to cope with stressors relate to disease and mortality risk, and lifestyle habits may be underlying mechanisms. Studies show psychological *symptoms* (eg, anxiety) and *states* (eg, happiness) predict the likelihood of adopting an integrated lifestyle that encompasses key health-related behaviors, like smoking. Yet, whether psychological *processes*, including stress-related coping, influence the adoption of a healthy lifestyle is unknown. We investigated whether coping strategies typically deemed adaptive (eg, seeking emotional support) and maladaptive (eg, denial) relate to sustaining a healthy lifestyle over a 16-year follow-up. We also explored whether variability in the use of these strategies, reflecting attempts to find the best strategy for a given stressor, subsequently relates to lifestyle.

**Methods:**

Women (*N* = 46 067) from the Nurses’ Health Study II cohort reported their use of 8 coping strategies in 2001, from which we also derived coping variability levels (lower, moderate, greater). Health behaviors (eg, physical activity, smoking, sleep), self-reported every 4 years from baseline until 2017, were combined into a lifestyle score. Generalized estimating equations, controlling for baseline demographics and health-related factors, were performed.

**Results:**

Most adaptive strategies and greater variability levels were associated with a higher likelihood of sustaining a healthy lifestyle (eg, active coping, relative risk [RR] = 1.09, 95% confidence interval [CI], 1.08-1.11), with the reverse evident with maladaptive strategies (eg, behavioral disengagement, RR = 0.94, CI, 0.93-0.95), but some unexpected results also emerged.

**Conclusions:**

Findings highlight the importance of going beyond the usual (mal)adaptive categorization of coping strategies when investigating their predictive value with behavioral outcomes.

## Teaser

Midlife women who cope more adaptively and flexibly with stressors are more likely to sustain healthy lifestyle behaviors up to 16 years later.

## Introduction

### Coping and health research

Early and recent studies indicate that strategies adults habitually use to cope with stressors, like planning actions or engaging in denial to handle a situation, are related to long-term physical health outcomes including longevity and chronic disease onset among initially healthy populations.^1–8^ Some of these findings also show stronger associations among women, possibly given their greater exposure to psychosocial stressors compared to men.^9^ Coping processes may affect long-term health outcomes partly because they influence behaviors that are proximal drivers of health (eg, tobacco smoking).^10,11^ For instance, individuals who disengage from stressful situations may be more likely to drink alcohol or smoke tobacco as a way to manage any accompanying distress, which over time can increase disease and mortality risk. Such behaviors tend to cluster,^12^ leading to a *lifestyle* that has a multiplicative effect on major health outcomes (eg, mortality risk) relative to the effects of engaging in any one behavior.^13^ To date, studies revealing associations between psychological factors and future lifestyle have mainly considered *negative psychological distress symptoms*, including anxiety and depression^14–16^ or *positive states and traits*, like happiness and optimism.^17–19^ Yet, whether psychological *processes* (ie, dynamic mechanisms that can modulate positive and negative psychological symptoms and states, such as stress-related coping) predict subsequent lifestyle is understudied. Prior work suggests these processes may, in fact, serve as important novel and alterable targets for primary prevention strategies aimed at promoting favorable lifestyles and healthy aging.^20,21^

### Commonly assessed coping strategies and variability in their use

Over 400 individual coping strategies have been documented in prior research.^22^ Yet, reviews of existing self-reported coping scales identified the following strategies as being the most assessed^23,24^: active coping, acceptance, seeking social support, reinterpretation, denial, and avoidance. These strategies are either cognitive (eg, reinterpretation) or behavioral (eg, active coping), which is consistent with the definition of coping which refers to cognitive and behavioral endeavors that aim to alter taxing demands from the environment.^25^ While other strategies were not as frequently assessed according to these reviews,^23,24^ they are likely to co-occur with the use of social support given their social nature (eg, engaging in religious activities, venting of emotions) or be conceptually intertwined with avoidance (eg, behavioral disengagement).^22^

Coping strategies are often viewed as being adaptive or maladaptive, as a function of their influence on mental and physical health outcomes.^24,26,27^ For example, meta-analytic findings showed that greater use of active coping and planning is related to higher psychological well-being levels, whereas greater use of denial and behavioral disengagement is related to higher anxiety and depression symptoms.^24^ However, any given strategy may not be inherently adaptive or maladaptive. In fact, some scholars have argued that the impact of coping strategies may depend on the flexibility with which they are used across contexts.^28,29^ This argument implies that optimal psychological adjustment to stressors will be evident among individuals who demonstrate *variability* in their selection and implementation of strategies to possibly maximize the appropriate use of specific strategies in any given context.^29^ Said differently, the variability with which individuals use coping strategies, that is to what extent one will consistently favor one strategy over others, use several strategies similarly, or alternate between a repertoire of various strategies, likely reflects their attempts to find the best strategy for a given situation.^29^

To assess various coping strategies, meta-analytic findings^24^ identified the coping orientation to problems experienced (COPE) inventory^26^ (and its shorter version, the Brief-COPE^30^) as the most commonly used coping scale. Despite some limitations (eg, low internal reliability on certain subscales), the COPE inventory remains widely used as it covers a breadth of cognitive and behavioral strategies, which are deemed either adaptive and maladaptive, and are believed to be relatively stable across different types of stressors.^24,26,30^ Limited but suggestive evidence using the COPE inventory also suggests that individual coping strategies and variability in their use are related to longevity and disease outcomes.^2,31,32^ Thus, research leveraging the COPE inventory and looking into coping’s role in the adoption of an overall healthy lifestyle, defined as an aggregate of health behaviors, appears promising.

### Coping and health behaviors

Conceptual frameworks in social epidemiology posit that coping strategies may affect disease and longevity outcomes in part via mechanistic behaviors that lie on the pathway between them, like tobacco smoking and physical activity.^10,33^ The theoretical *Strength Model of Self-Control*^34^ in psychology also supports a linkage between coping and health behaviors. This model postulates that self-regulation, the process by which one alters their thoughts, emotions, and behaviors, relies on limited resources. As a result, people with many demands on their self-regulatory system tend to be less successful in regulating across domains, including health behaviors.^34,35^ Empirical results support this idea: for instance, adults from a low-income community reported that, although they knew habits like smoking and alcohol drinking were detrimental, their willpower to resist engaging in them was depleted when they had to cope with many stressors on a given day.^36^ This model suggests people who favor adaptive coping strategies (eg, active coping) may deal more efficiently with stressors and thereby have more resources (eg, energy, time) to adopt favorable lifestyles which over time lead to better health outcomes. In contrast, people who favor maladaptive strategies (eg, denial) may not handle stressors as efficiently, thus depleting their available resources to engage in favorable lifestyles.

Several studies, mostly cross-sectional, have examined if distinct strategies used to cope with stressors are related to specific health behaviors. These studies found greater use of strategies usually viewed as adaptive, like planning ahead and drawing on religious beliefs/behaviors, was related to better diet quality, more physical activity, and smoking abstinence,^37–39^ while greater use of strategies usually deemed maladaptive, like denial and suppressing one’s emotion, was linked to poorer sleep and diet quality, less physical activity and heavy alcohol drinking.^37,38,40^ Yet, cross-sectional studies prevent conclusions about the directionality of effects, which is problematic since behaviors may also impact how one subsequently copes with stressors.^41,42^

Prospective evidence on the linkages of coping with multiple concurrent health behaviors is scarce. To the best of our knowledge, several longitudinal studies have focused on one behavior at a time (eg,^43,44^), but only one evaluated many behaviors in the same sample.^45^ Among 565 African Americans followed over a 2.5-year period, greater use of religious beliefs/behaviors to cope with stressors was unrelated to future diet quality, alcohol intake, smoking status, and physical activity assessed separately.^45^ Yet, it remains unclear if (1) religious coping is related to separate health behaviors in other populations; (2) religious coping is associated with the adoption/maintenance of many concurrent health behaviors aggregated into a lifestyle score; (3) other stress-related coping strategies (eg, active coping, self-blame) and variability in their use may relate to future behaviors (individuals or aggregated); and (4) these relations persist over a longer follow-up period.

### The current study

In this study, we examined whether 8 individual coping strategies and variability in their use at the study baseline were each related to the likelihood of sustaining a healthy lifestyle over a 16-year period. Associations were evaluated in disease-free women, given that prior work showed stronger linkages of coping with disease incidence in women^9^ and that chronic diseases (eg, heart disease, cancer) can alter both the use of coping strategies and the adoption or maintenance of healthy behaviors.^46,47^ Based on previous work,^2,26,27^ we hypothesized that the likelihood of sustaining a healthy lifestyle would be higher with greater use of adaptive strategies (eg, emotional support) and lower with greater use of maladaptive strategies (eg, denial). We further explored the association of coping variability—operationalized as the extent to which strategies are (un)equally used, in general^2,32^—with such a sustained healthy lifestyle without an *a priori* hypothesis, given limited work on this relationship. Following prior research,^2–6^ we considered baseline demographics (eg, age) and health-related factors (eg, body mass index) as potential confounders. Secondary analyses assessed coping’s role in the maintenance of each health behavior separately, with similar hypotheses as those posited for the aggregated lifestyle score.

## Methods

### Participants

The Nurses’ Health Study II is an ongoing cohort study that was launched in 1989 among 116 429 female nurses aged 25-42 years. Participants completed biennial questionnaires on lifestyle, medical history, and newly diagnosed conditions, with response rates of >85% across cycles.^48^ The coping measure was administered in 2001, which constitutes the current baseline, as part of a substudy on violence exposure in a subset of participants (*n* = 68 365). Exclusions, mainly due to missing data, led to an analytic sample of 46 067 (Supplementary Figure S1). Eligible versus non-eligible participants based on their completion of the coping items specifically were less likely to have a healthy diet score but otherwise were highly similar (Supplementary Table S1). The study protocol was approved by the IRBs of the Brigham & Women’s Hospital/Harvard T.H. Chan School of Public Health (#1999P003389), the Université du Québec à Trois-Rivières (#CERPPE-22-04-10.05) and the Centre intégré universitaire de santé et de services sociaux de l’Est-de-l’Île-de-Montréal (#2022-2968).

### Measures

#### Coping

Details about the coping measure and the current dispositional coping variability construct are provided in Supplementary Text S1.

How individuals *typically* cope with stressful events, as a dispositional style, was measured once in 2001 using a modified version of the validated self-report 60-item Coping Orientation to Problems Experienced (COPE) inventory.^26^ The current version encompassed 8 subscales representing commonly used coping strategies. Four subscales reflect strategies deemed adaptive: active coping, use of emotional support, acceptance, and religion; another 4 subscales capture strategies deemed maladaptive: denial, behavioral disengagement, focus on and venting of emotions, and self-blame.^26^ Each subscale includes 2 items rated on a scale from 0=*“Not at all*” to 3=*“A lot*” that were combined to derive a score ranging from 0 (less frequent use) to 6 (more frequent use). Continuous scores from subscales were standardized using *z*-scores to ease comparisons with prior work. Participants with missing data on any item were excluded.

Following recent work,^2,32,49^ we used this single assessment to derive the dispositional between-strategy index (SD_between_) that captures the variability one *typically* has in the use of their coping strategies. This SD_between_ reflects the amount of variation in the frequency of use of the 8 coping strategies. Individuals with higher variability levels display frequency scores that are highly uneven across the 8 coping strategies; thus, they are more likely to select and rely on one or a few strategies only and discard others when handling stressors. Conversely, those with lower variability levels display frequency scores that are highly similar across the 8 coping strategies; thus, they are more likely to use all or nearly all strategies to a similar extent across situations. Lastly, individuals scoring in the moderate range display frequency scores that are only moderately uneven across the 8 coping strategies; thus, they tend to use most or all strategies but to varying extent depending on the strategy, perhaps reflecting efforts to find the best fit or prioritize a given strategy for a given context.^49^ Thus, moderate (vs. greater or lower) variability is conceptualized as the most flexible way of coping with stressors. Accordingly, prior work has shown that lower and higher variability levels, relative to moderate levels, are related to poorer psychological and longevity outcomes.^32,50^ Such non-linear linkage is also analogous to the association of vagally mediated heart rate variability with psychological outcomes, whereby lower and higher (vs moderate) variability levels in this measure of the parasympathetic nervous system have been related to greater depressive symptoms and lower positive affect in women.^51^

To investigate such potential discontinuity effects,^29^ we tertiled the dispositional Between-Strategy Index (lower, moderate, greater levels). Of note, characterizing coping variability with a standard deviation (SD) score can be confounded by the average level of strategies favored^49^; namely, individuals with consistently low or high mean levels in frequency of use across strategies cannot display high variability levels due to floor/ceiling effects. Therefore, following prior work,^2,32,49^ we further controlled for the mean level of frequency of endorsing strategies used at baseline in all models.

#### Lifestyle score

The 5 health behaviors included in the lifestyle score were physical activity, diet quality, alcohol and tobacco consumption, and sleep quality/duration, selected based on a lifestyle index of multiple health behaviors commonly used in prior studies,^16,19,52,53^ as well as disease prevention guidelines.^54–56^ Data on physical activity, diet, alcohol, and tobacco consumption were collected via self-report at baseline (2001), and every 4 years until 2017, whereas data on sleep quantity/quality were obtained in 2001, 2013, and 2017. Thus, we ran analyses with and without sleep as a component of the healthy lifestyle score.

We dichotomized each behavior based on whether women met the recommended guidelines at each follow-up assessment (1 = yes; 0 = no). Physical activity was measured with a validated questionnaire, which showed high correlations with activity reported on past-week activities recalls and 7-day activity diaries^57^; a score of 1 was assigned when women reported ≥ 150 minutes per week of moderate-to-vigorous activity (eg, brisk walking, running, bicycling). Women reported their current smoking status (current, former, or never smoker), which was found to be highly correlated with toenail nicotine levels^58^; they received a score of 1 if they reported being a current non-smoker. Dietary information was obtained from the 131-item Food Frequency Questionnaire, which has high reproducibility and validity when compared with 1-week diet records over a one-year period.^59^ Diet score encompasses the Alternative Healthy Eating Index (AHEI), a revised version from the US Department of Agriculture Healthy Eating Index^60^ and incorporates: a higher intake of vegetables, fruit, whole grains, nuts, and legumes, long-chain (n-3) fatty acids, polyunsaturated fats; lower intake of sugar-sweetened beverages and fruit juice, red/processed meat, saturated fats, sodium. The conventional score for each dietary component ranges from 0 (worst dietary behavior) to 10 (optimal dietary behavior), and then scores were summed. A healthy diet was defined as a total score in the top 40% of the current sample distribution, updated at each time point, since this cutoff strongly relates to a lower risk of several diseases, including diabetes and cancer, in this cohort.^61^ Alcohol consumption was noted as the monthly-to-daily frequency of alcoholic beverage intake (eg, beer, wine, liquor). The role of alcohol in health is complex, with findings showing detrimental and protective effects on disease outcomes.^62,63^ Moderate intake versus abstinence has been related to a lower risk of certain diseases,^62,64^ which may be attributable to physiological or social benefits of moderate use. Yet, other evidence suggests the detrimental linkage with abstinence may be explained, at least in part, by pre-existing poor health among those who stopped drinking as they became unwell.^64^ Accordingly, it is increasingly argued that no level of alcohol intake is health-beneficial.^65,66^ Thus, and following recent disease prevention guidelines,^67,68^ healthy alcohol consumption was herein coded as drinking 0 drinks/day on average, while drinking > 0 drinks/day on average was coded as unhealthy. Lastly, sleep was characterized by single items on quality and/or duration, whenever the information was available (ie, 2001, 2013, and 2017). In 2001, only sleep duration was available (ie, <5 hours, 5, 6, 7, 8, 9, ≥10 hours), which has been found highly correlated with sleep diaries^69^; a score of 1 was given for 7-8 hours/night. In 2013, only sleep quality/difficulty items were queried (ie, rarely/never has difficulty falling asleep, unintentionally waking up during the night, and waking up too early and not being able to fall back asleep = 1; else = 0). In 2017, both sleep duration and quality/difficulty were assessed (categorization like 2001 and 2013 assessments) and were combined to define healthy sleep (1 = healthy sleep duration and no sleep difficulty) versus everything else (0 = unhealthy sleep duration and/or present sleep difficulties).

Binary scores from each health behavior were then summed to create the lifestyle score, ranging from 0 “least healthy” to 4 or 5 “most healthy,” which was updated every 4 years, for a total of 5 lifestyle assessments including the one in 2001. Because a score of 4 or 5 (healthy lifestyle) vs a score of 0-3 (unhealthy lifestyle) was associated with ~50% decreased risk of stroke in this cohort^55^ and 66% decreased risk of mortality in prior research,^13^ we defined a *healthy lifestyle* using this cutpoint (endorsing ≥4 healthy behaviors; yes/no) in the secondary analyses including sleep; as primary analyses were based on 4 rather than 5 behaviors, we used the cutpoint of endorsing ≥3 healthy behaviors. Women with missing data on baseline or all follow-up lifestyle assessments, as defined by >2 behaviors missing,^16,19^ were excluded. Of note, the vast majority of the analytic sample completed all 5 lifestyle assessments at baseline (*n* = 45 334); the remaining women who had completed some assessments (*n* = 733) were less likely to have healthy BMI and more likely to have healthy alcohol consumption levels, but otherwise were remarkably similar to those with all follow-up lifestyle information on demographic, medical, behavioral, and psychological characteristics at baseline (Supplementary Table S2).

Following prior work,^19^ we further defined *sustained healthy lifestyle* as reporting a healthy lifestyle score at least twice over the study period, including the baseline assessment. Approximately half of the analytic sample reported sustaining a healthy lifestyle (≥2 healthy lifestyle scores) over the study duration (without the sleep component: 55.1%; with the sleep component: 48.6%). Among those, most did so on 2 consecutive lifestyle assessments (eg, had a healthy lifestyle score in 2005 and 2009; without the sleep component: 83.1%; with the sleep component: 97.3%).

#### Covariates

Selected confounders and other covariates were self-reported at the 2001 baseline unless otherwise noted. Demographic factors included age (continuous), race (White, non-White; reported in 1989), census tract income (continuous), and marital status (married/in a relationship, divorced/separated/widowed). Health-related factors included body mass index (BMI; <25 kg/m^2^, ≥25 kg/m^2^) and having a physical exam in the last 2 years (yes, no), as a proxy for a context where individuals may receive advice from a clinician that is associated with lifestyle changes.^70^ Participants with missing data on any item were excluded.

### Statistical analysis

#### Descriptive statistics

All statistical analyses were conducted using SAS v9.4. We first calculated the means and standard deviation (SD) or frequencies for each covariate within the analytic sample (*N* = 46 067) and across coping variability levels. We then computed Pearson and Spearman correlations across the scores of the 8 COPE subscales and 3 variability levels to evaluate the associations between individual strategies and the variability scores. Lastly, to characterize the degree of change in lifestyle over time, within-subject coefficients of variation (CVs) were computed.^71^

#### Primary models

Associations of baseline individual coping strategies and variability in their use with the likelihood of reporting a sustained healthy lifestyle over the follow-up period were evaluated using separate generalized estimating equation (GEE) models to account for correlated observations; further, a Poisson distribution was used to account for non-rare outcomes.^72^ In primary models, we evaluated each individual coping strategy measured continuously (standardized; per 1-SD) and coping variability categories (lower, moderate, higher levels) in relation to having a sustained healthy lifestyle (with and without sleep scores), separately. Models controlled for age, race, census tract income, marital status, BMI, and recent physical exam.

#### Secondary models

We performed 2 sets of secondary analyses. First, to quantify the influence of unmeasured confounders, we calculated the *E*-value, defined as the minimum strength of association that an unmeasured confounder would need to have with both exposure and outcome to fully explain an observed association.^73^ Second, to evaluate if associations were consistent across behaviors, we investigated the likelihood of sustaining a healthy level of each behavior.

## Results

### Baseline characteristics

At baseline, participants were 46.2 years old on average (SD = 4.7; range = 36-55) and with a mean census tract income of 66K (SD = 24K; **Table 1**). They were mostly White (97.3%; Asian [1.2%], Black [1.0%], Native American [0.4%], and Hawaiian [0.1%]) and married/in a relationship (80.8%). Although most were not current smokers and reported having a recent physical exam and being good sleepers, only a third to a half had a healthy BMI (<25 kg/m^2^) and favorable levels of physical activity, diet quality, and alcohol intake. When considering the distribution of these characteristics across coping variability levels (**Table 1**), participants with greater versus lower levels were less likely to have a healthy BMI but more likely to have favorable levels of physical activity, diet quality, and sleep; they did not differ on demographics.

**Table 1:** Distribution of covariates and health behaviors in the overall sample and according to coping variability levels^§^ in 2001.

		Coping variability levels		
	Total (*n* = 46 067)	Lower (*n* = 15 171)	Moderate (*n* = 15 520)	Greater (*n* = 15 376)
*Demographic characteristics*				
Age, M (SD)	46.2 (4.7)	46.1 (4.6)	46.2 (4.7)	46.3 (4.7)
White^*^, %	97.3	97.3	97.1	97.4
Married/in a relationship, %	80.8	79.1	80.9	82.2
Census tract income in thousands of dollars, M (SD)	66 (24)	66 (24)	66 (24)	66 (24)
				
*Health-related factors*				
Body mass index < 25 kg/m^2^, %	50.5	47.0	51.2	53.2
Physical exam in last 2 years, %	87.5	86.2	87.5	88.7
				
*Health behaviors*				
Current non-smoker	92.4	91.1	92.5	93.5
≥150 min/week of moderate-to-vigorous physical activity, %	45.2	41.0	45.3	49.3
Favorable AHEI diet score (in top 40 percent), %	40.2	36.7	40.9	42.8
Alcohol intake 0 drink/day, %	38.7	36.9	37.9	41.4
Sleep 7-8 h/night^**^, %	67.2	64.6	67.8	69.1

*Notes.* Values are means (SD) for continuous variables and percentages for categorical variables. Values of polytomous variables may not sum to 100% due to rounding.

^§^There were too many (ie, 8) individual coping strategies assessed to present covariates’ distribution by each of them.

^*^Because the non-White category represents only 2.7% of the total sample, its subcategories—namely Asian (1.2%), Black (1.0%), Native American (0.4%), and Hawaiian (0.1%) – were too small and could not be studied separately.

^**^Sleep was characterized with quality and/or duration data, whenever the information was available (ie, 2001, 2013, and 2017). Descriptives presented here are from the 2001 assessment, for which only sleep duration (7-8 h/night = 1 [healthy]; else = 0 [unhealthy]) was available. Abbreviation: AHEI = Alternative Healthy Eating Index.

The magnitude of correlations across scores from coping strategies varied but ranged from weak to moderate (|*r*|=0.01-0.53; Supplementary Table S3). Adaptive coping strategies were generally inversely and modestly correlated with maladaptive ones, reinforcing the idea that they are independent of one another as suggested in prior work.^26^ Correlation coefficients between coping variability and individual strategies were also null-to-moderate. Within individuals, lifestyle scores were stable over time (without sleep: CV = 0.27, 95% confidence interval [CI], 0.27-0.27; with sleep: CV = 0.29, 95%CI, 0.29-0.29).

### Associations of coping with healthy lifestyle over time

**Table 2** shows the associations of baseline coping exposures with the likelihood of sustaining a healthy lifestyle, without the sleep component. In age-adjusted models (Model 1), each 1-SD increase in the use of adaptive coping strategies was related to greater likelihood (eg, active coping, RR = 1.11, 95%CI, 1.09-1.12). Conversely, each 1-SD increase in the use of maladaptive strategies was related to lower likelihood, after adjusting for age (eg, behavioral disengagement, RR = 0.93, 95%CI, 0.92-0.94), except for a small greater likelihood with focus on and venting of emotions (RR = 1.02, 95%CI, 1.00-1.03). Compared to lower coping variability levels, moderate and higher levels were related to 17% and 30% greater likelihood, respectively, in age-adjusted models. Estimates were robust to further adjustment for demographic factors (Model 2) and health-related factors (Model 3), and notably similar when adding the sleep component (Table 3).

**Table 2:** Relative risk and 95% confidence interval of having a sustained healthy lifestyle (based on 4 components^†^) associated with the adoption of coping individual strategies and variability levels.

	Model 1: age RR (95% CI)	Model 2: demographics RR (95% CI)	Model 3: demographics + health-related factors RR (95% CI)	*E*-value (using Model 3 estimates)
*Individual coping strategies (per 1-SD increase)*				
Active coping	1.11 (1.09, 1.12)^***^	1.10 (1.09, 1.12)^***^	1.09 (1.08, 1.11)^***^	1.40
Acceptance	1.04 (1.03, 1.05)^***^	1.04 (1.03, 1.05)^***^	1.04 (1.03, 1.05)^***^	1.24
Religion	1.08 (1.07, 1.09)^***^	1.09 (1.08, 1.11)^***^	1.09 (1.08, 1.10)^***^	1.40
Use of emotional support	1.07 (1.06, 1.08)^***^	1.07 (1.06, 1.08)^***^	1.06 (1.05, 1.07)^***^	1.31
Focus on and venting of emotions	1.02 (1.00, 1.03)^**^	1.01 (1.00, 1.02)^*^	1.01 (1.00, 1.02)^*^	1.11
Denial	0.95 (0.94, 0.96)^***^	0.95 (0.94, 0.96)^***^	0.95 (0.94, 0.96)^***^	1.29
Behavioral disengagement	0.93 (0.92, 0.94)^***^	0.93 (0.92, 0.94)^***^	0.94 (0.93, 0.95)^***^	1.32
Self-blame	0.95 (0.94, 0.96)^***^	0.95 (0.94, 0.96)^***^	0.95 (0.94, 0.96)^***^	1.29
*Variability in coping strategies used*				
Moderate vs lower variability	1.17 (1.14, 1.20)^***^	1.17 (1.14, 1.20)^***^	1.15 (1.12, 1.19)^***^	1.57
Greater vs lower variability	1.30 (1.26, 1.33)^***^	1.29 (1.26, 1.33)^***^	1.27 (1.24, 1.30)^***^	1.86
Greater vs moderate variability	1.11 (1.08, 1.13)^***^	1.11 (1.08, 1.13)^***^	1.10 (1.07, 1.13)^**^	1.43

*N* = 46 067, *n*_events_ = 19 904 (where events = individuals who had a healthy lifestyle score at ≥2 time points over the follow-up period; number of events per variability levels: lower = 5670, moderate = 6784, greater = 7450).

^†^Healthy lifestyle components include diet, alcohol consumption, smoking status, and physical activity. The first 4 individual coping strategies are typically considered more adaptive whereas the last 4 strategies are typically deemed less adaptive. Although individual coping strategies and coping variability levels are presented in the same table, they represent distinct analyses.

^*^ *p*≤ .05;

^**^ *p* ≤ .01;

^***^ *p* ≤ .001.

Model 1 adjusted for age. Model 2 adjusted for age, race, census tract income, and marital status. Model 3 (core) adjusted for Model 2 as well as body mass index and physical exam within the last 2 years. All coping variability models further adjusted for mean of all individual coping strategies.

Abbreviations: CI = confidence interval, RR = relative risk, SD = standard deviation.

**Table 3:** Relative risk and 95% confidence interval of having a sustained healthy lifestyle (based on 5 components^†^) associated with the adoption of coping individual strategies and variability levels.

	Model 1: age RR (95% CI)	Model 2: demographics RR (95% CI)	Model 3: demographics + health-related factors RR (95% CI)	*E*-value (using Model 3 estimates)
*Individual coping strategies (per 1-SD increase)*				
Active coping	1.12 (1.10, 1.13)^***^	1.11 (1.10, 1.13)^***^	1.10 (1.09, 1.12)^***^	1.43
Acceptance	1.03 (1.02, 1.05)^***^	1.04 (1.02, 1.05)^***^	1.03 (1.02, 1.05)^***^	1.21
Religion	1.10 (1.08, 1.11)^***^	1.11 (1.09, 1.12)^***^	1.10 (1.09, 1.12)^***^	1.43
Use of emotional support	1.09 (1.08, 1.10)^***^	1.09 (1.08, 1.10)^***^	1.08 (1.07, 1.10)^***^	1.37
Focus on and venting of emotions	1.02 (1.01, 1.03)^**^	1.02 (1.00, 1.03)^**^	1.02 (1.00, 1.03)^**^	1.16
Denial	0.95 (0.94, 0.96)^***^	0.95 (0.94, 0.96)^***^	0.95 (0.94, 0.97)^***^	1.29
Behavioral disengagement	0.93 (0.91, 0.94)^***^	0.93 (0.92, 0.94)^***^	0.94 (0.92, 0.95)^***^	1.32
Self-blame	0.94 (0.93, 0.95)^***^	0.94 (0.93, 0.95)^***^	0.95 (0.93, 0.96)^***^	1.29
*Variability in coping strategies used*				
Moderate vs lower variability	1.19 (1.16, 1.23)^***^	1.19 (1.16, 1.23)^***^	1.18 (1.14, 1.21)^***^	1.64
Greater vs lower variability	1.33 (1.29, 1.36)^***^	1.32 (1.28, 1.36)^***^	1.30 (1.26, 1.34)^***^	1.92
Greater vs moderate variability	1.11 (1.08, 1.14)^***^	1.11 (1.08, 1.14)^***^	1.10 (1.07, 1.13)^***^	1.43

*N* = 46 067, *n*_events_ = 17 678 (where events = individuals who had a healthy lifestyle score at ≥2 time points over the follow-up period; number of events per variability levels: lower = 4958, moderate = 6054, greater = 6666).

^†^Healthy lifestyle components include diet, alcohol consumption, smoking status, physical activity, and sleep. The first 4 individual coping strategies are typically considered more adaptive whereas the last 4 strategies are typically deemed less adaptive. Although individual coping strategies and coping variability levels are presented in the same table, they represent distinct analyses.

^*^ *p*≤ .05;

^**^ *p* ≤ .01;

^***^ *p*≤ .001.

Model 1 adjusted for age. Model 2 adjusted for age, race, census tract income, and marital status. Model 3 (core) adjusted for Model 2 as well as body mass index and physical exam within the last 2 years. All coping variability models further adjusted for a mean of all individual coping strategies. Abbreviations: CI = confidence interval, RR = relative risk, SD = standard deviation.

### Potential residual confounding

**Tables 2–3** also report *E*-values obtained for each association, after adjusting for demographics and health-related factors (Model 3). Coefficients vary from 1.11 to 1.86 in models with the lifestyle score excluding sleep and were slightly stronger, ranging from 1.16-1.92, after including sleep in the score. These values suggest that the observed estimates could be explained away only by an unmeasured confounder that was related, beyond the measured covariates, to both coping exposures and a sustained lifestyle with a risk ratio (*E*-value) of 1.11- to 1.92-fold each.

### Associations of coping with individual healthy behaviors over time

Supplementary Tables S4-8 show the associations of baseline coping exposures with the likelihood of sustaining each behavior separately. Adaptive strategies as well as moderate and greater (vs. lower) variability levels were generally related to a greater likelihood of sustaining healthy diet, physical activity, and sleep levels, whereas maladaptive strategies were related to lower likelihood. Yet, smoking and alcohol results stood out (**Supplementary Tables S4**  **and**  **S7**). For smoking (and to a lesser extent, alcohol), most estimates were of small magnitude, but they were statistically significant, with narrow confidence intervals, and in the expected directions (Supplementary Table S4). This pattern is likely due to the low prevalence of smoking in the sample (<10% at baseline). Some estimates for alcohol were somewhat unexpected (Supplementary Table S7). For instance, greater use of active coping and use of emotion support were related to a *lower* likelihood, whereas greater use of behavioral disengagement was related to a *higher* likelihood of sustaining healthy consumption over time.

## Discussion

This study examined whether strategies women generally use to cope with stress were associated with likelihood of sustaining a healthy lifestyle over 16 years. Findings indicated that women reporting more use of strategies typically viewed as adaptive, like actively coping with stressors and using emotional support, were 3%-10% more likely to report a healthy lifestyle at least twice throughout the study. Conversely, more use of strategies usually deemed maladaptive, like denying stressors and behaviorally disengaging from them, was related to a 5%-6% lower likelihood of sustaining a healthy lifestyle. Showing variability in the use of these strategies also appeared beneficial, as moderate and greater coping variability levels (vs. lower ones) were associated with 18%-30% greater likelihood of sustaining a healthy lifestyle over a 16-year follow-up period.

Overall, these findings are consistent with our hypotheses, prior conceptual theories and models,^10,33,34^ as well as previous cross-sectional results on single behaviors.^37–40^ They are further aligned with prior longitudinal evidence linking psychological distress and positive states/traits with reporting a healthy lifestyle over many years.^16,19,53^ Moreover, associations remained evident after statistical control for baseline demographics and health-related factors, with *E*-values suggesting that residual unmeasured confounding was unlikely to be substantial, at least for several strategies (eg, active coping, religion, use of emotional support, behavioral disengagement) as well as moderate and greater variability levels. While some effects were small, it is worth remembering that they add to the initial risk already incurred (and may even be conservative estimates) as the sample comprised women who were already at midlife.

Some findings were unexpected, such as the beneficial effect of focusing on and venting emotions when facing stressors, a strategy that is usually considered maladaptive. As with other coping subscales,^23,24^ 2 coping strategies are captured in this subscale. While focusing on and ruminating about stressors and related emotions is usually detrimental to health outcomes,^24,74^ prior work has found venting emotions may have positive effects, particularly when used in an emotionally-receptive social context.^75,76^ Thus, women in this sample may generally have nourishing and emotionally receptive relationships; besides gaining benefit from venting in this context, having supportive relationships may also help women cope more actively with stressors and, in turn, promote healthier behaviors. Reinforcing this speculation is our finding that active coping and the use of emotional support were the 2 strategies most strongly correlated with focusing on and venting emotions, and also consistently related to a greater likelihood of sustaining a healthy lifestyle. Another unexpected result was the *lower* likelihood of sustaining healthy alcohol consumption with greater use of emotional support, a strategy typically judged as adaptive. It is plausible that women socially drink alcohol when they gather with close ones to handle stress. Aligned with the premise of the coping flexibility framework,^28,29^ these results reinforce the idea that focusing on and venting emotions may not be inherently maladaptive and using emotional support may not be inherently adaptive, but their impact—at least on health behaviors—may in fact depend on the social context.

To the best of our knowledge, this is the first study exploring the association of coping variability with future lifestyle and behaviors. The monotonic relation of coping variability with health outcomes—whereby more variability predicts more favorable outcomes—has been debated.^29^ Here, midlife women reporting moderate or greater variability levels were more likely to sustain a healthy lifestyle. At first, these findings appear inconsistent with those of prior health research conducted in the Midlife in the US study (MIDUS), which used the same variability algorithm and showed shorter (rather than longer) lifespans for participants with the highest coping variability levels.^32^ Nonetheless, the current findings are aligned with recent research also obtained in the Nurses’ Health Study II and relying on the same variability algorithm, which found that moderate and greater (vs. lower) variability levels were related to gains in lifespan.^2^ As posited by the authors,^2^ differences in patterns between these 2 studies may be because participants in the greater coping variability level had more extreme discrepancies across scores in the strategies they used (ie, certain strategies were similarly highly used and these were at the clear expense of others) in the MIDUS compared to the Nurses' Health Study II, hinting possibly to less flexible attempts to cope with stressors. In other words, results from these studies imply that more dispositional coping variability may be advantageous for longevity and behavioral outcomes, but only to a certain level.

One underlying mechanism of the coping variability-lifestyle linkage may be coping adequacy, which may unfold from coping more flexibly with stressors: recent results indeed show that adults who judged their coping as more adequate have a healthier lifestyle.^77^ Aligned with the *Strength Model of Self-Control*,^34^ those who cope more flexibly may also be more efficient at handling stressors and, in turn, have more capacity for engaging in healthy behaviors. Another plausible explanation is that more flexible individuals may, in fact, use healthy behaviors as coping strategies per se. For instance, one could engage in physical activity when strategies like active coping or emotional support are less adequate or unavailable at a given moment. Future studies on the coping-lifestyle linkages should seek replication with alternative variability indices^29^ and must disentangle behaviors used as habits versus regulatory processes.^41,42^

This study has some limitations. First, we did not have information about stressful events that serve as the context for coping, which can vary in nature, number, intensity, and chronicity, as well as across participants. It is possible that these characteristics influence the selection and implementation of coping strategies and variability in their use. However, previous research with the COPE inventory found moderate-to-high concordance between the dispositional and situational (ie, across distinct contexts/stressors) versions of the inventory.^26,78^ Moreover, prior findings on the coping-health linkage indicated similar results when stressors were imposed by researchers versus selected by participants,^27^ suggesting that even if our participants reflected on stressors of different nature, intensity, or chronicity when answering the coping items, it is unlikely to drive consequential variations. Likewise, a one-time assessment of how individuals generally cope with stressors, as captured by the COPE inventory, may be influenced by many factors, including memory bias. It also does not capture contextual features that can alter coping strategies usage (eg, with whom, where, and when in the day/week the person is). Yet, it is worth noting that prior studies looking at variability in the use of such strategies documented a clear correspondence between dispositional between-strategy variability (captured at a single time assessment, as in the current study) and within-strategy variability (captured across multiple daily assessments, hence lowering memory issues and allowing nuanced consideration of contextual features; meta-analytic *r = *.47, *p* < .001).^49^ Altogether, a one-time general assessment of coping strategies usage may be a reasonable proxy of how individuals cope with stressors on a daily basis and across contexts.

Second, self-reported habits may be vulnerable to social desirability, which can lead to biased estimation of health behaviors.^79^ Yet, even if absolute levels are not reported precisely, behaviors would likely still be categorized appropriately as either healthy/unhealthy. Besides, and as mentioned earlier, the intricate alcohol-health linkage likely varies as a function of both alcohol frequency (eg, abstainer, moderate, or heavy drinkers) and history (eg, lifetime abstainers vs. former drinkers). An in-depth investigation of how coping relates to various profiles of alcohol consumption was beyond the scope of the current lifestyle study using a binary scoring approach and should be examined in future research. Lastly, findings were from a sample of mainly White, middle-aged female nurses free of chronic disease at baseline. While results on behavioral factors from occupational cohorts are usually comparable to those in the general population,^80^ nurses may cope with stressors more adaptively and display greater health-related conscientiousness (eg, show self-discipline towards lifestyle factors) than other women in the general population.^81,82^ Thus, investigating these associations among more diverse occupational and racial/ethnic samples is warranted, because the ways other populations cope with stressors may not be as adaptive and flexible and, in turn, relate more strongly, or at least, differently, to lifestyle factors.

Strengths of the study include its prospective design over more than a decade, in a large and richly characterized sample. Further, the use of a validated coping scale including many strategies allowed the comparison of their respective predictive value for the maintenance of a healthy lifestyle, which points to specific potential intervention targets. We also acknowledged the complexity of coping and moved beyond the traditional categorization of adaptive versus maladaptive strategies by exploring if variability in their use relates to lifestyle maintenance.

### Conclusion

Altogether, findings indicate that the maintenance of a healthy lifestyle over many years may be promoted by the greater use of various adaptive strategies to cope with stressors but impeded by the greater use of many maladaptive coping strategies. Beyond the inherent (mal)adaptive nature of these strategies, greater variability in their use, possibly reflecting flexibility in how they are selected and implemented across situations, also predicted a higher likelihood of sustaining a healthy lifestyle. In recent years, behavioral medicine and public health leaders have urged scientists to consider markers of positive psychological functioning, including the capacity to cope effectively with stressors, as novel and alterable determinants of long-term physical health and related behaviors.^20,21^ Psychotherapies also increasingly favor a transdiagnostic approach that directly tackles processes like coping strategies and promotes their flexible use,^83^ potentially a more cost-effective method of treatment than intervening upon stressors, psychological symptoms, and states separately to improve lifestyle habits. Hence, supporting adults to cope adaptively and flexibly with stressors may be an innovative way to free up energy and time resources that can be redirected towards the adoption of a healthy lifestyle over time. Considering these coping processes as modifiable determinants of subsequent lifestyle, especially in future clinical trials, would be aligned with current scientific guidance and clinical efforts.

## Supplementary Material

kaaf006_suppl_Supplementary_Figures_1_Tables_1-8_Texts_1

## Data Availability

De-identified data from this study are not available in an a public archive. Procedures to obtain and access data from the Nurses’ Health Studies is described at https://www.nurseshealthstudy.org/researchers (contact email: nhsaccess@channing.harvard.edu).

## Transparency statements

*Study registration*: This study was not formally registered; Analytic plan registration: This analytic plan was not formally pre-registered. *Analytic code availability*: Analytic code used to conduct the analyses presented in this study are not available in a public archive. They may be available by emailing the corresponding author. *Materials availability*: Study materials are available at: https://www.nurseshealthstudy.org/participants/questionnaires. *Ethics approval*: The study protocol was approved by the Institutional Review Boards of the Brigham and Women’s Hospital and the Harvard T.H. Chan School of Public Health (#1999P003389), Université du Québec à Trois-Rivières (#CERPPE-22-04-10.05) and Centre Intégré Universitaire de Santé et Services Sociaux de l’Est-de-l’Île-de-Montréal (#2022-2968).
