# Supplementary material for: Are the ways women cope with stressors related to their health behaviors over time?
Source: Ann Behav Med. 2025 Feb 6;59(1):kaaf006. doi: 10.1093/abm/kaaf006 (PMC11799860; doi:10.1093/abm/kaaf006)
Supplement: kaaf006_suppl_Supplementary_Figures_1_Tables_1-8_Texts_1 [file kaaf006_suppl_supplementary_figures_1_tables_1-8_texts_1.docx]

**Supplemental Figure 1.** Flowchart of the analytic sample.


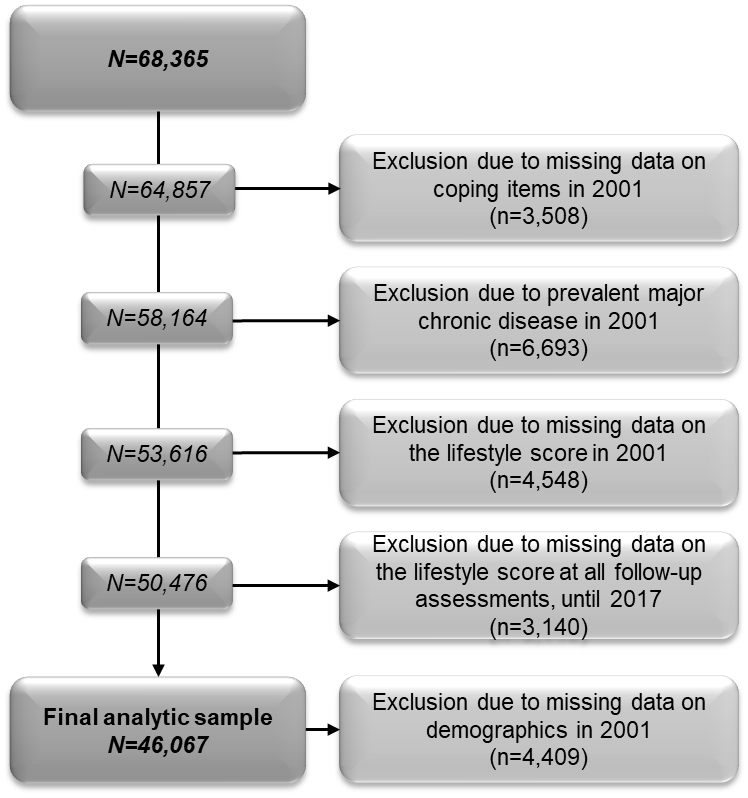


**Supplemental Text 1.** Additional details about the coping measure and the dispositional coping variability construct*.*

Computing Cronbach Alpha coefficients (α) based on 2-item measures is not informative [1]. However, the original COPE scale, which relies on a greater number of items per subscale, has shown strong psychometric properties among University students: the validation study reported moderate-to-excellent alpha values for internal consistency, ranging from α=0.62 to 0.92, and test-retest correlations across 2 months ranging from r =0.48 to 0.86 for the same subscales administered in the Nurses’ Health Study II cohort (e.g., Acceptance, Self-Blame) [2]. In another sample of U.S. adults from the general population, 4-item coping subscales of the COPE inventory had acceptable-to-good internal consistency reliability, ranging from α=0.65 to 0.79 [3]. Scholars have previously noted that coping scales tend to have somewhat lower internal consistency values compared to those of other psychological measures [4-6], possibly because some subscales combine items that capture more than one strategy (e.g., Focus on & Venting of Emotions). Given the lack of consensus about how subscales should be combined to define coping styles subsuming multiple strategies (e.g., problem- versus emotion-focused), because of empirical and theoretical reasons explained elsewhere [3] and consistent with the latest recommendations regarding use of the COPE inventory [7], we considered the eight coping subscales separately.

Coping variability has been conceptualized in diverse ways. For instance, an earlier approach referred to the “repertoire,” whereby the sum of more (versus less) used strategies represents the breadth of strategies one can use to cope with stressors, with a higher score indicating a broader coping repertoire [8, 9]. Conversely, recent research introduced and validated the “Between-Strategy Index”, a standard deviation algorithm that characterizes differences between the score related to frequency of use for each strategy and the respective mean frequency score across all strategies [10]. More specifically, between-strategy variability indicates the extent to which each strategy is generally used, on the full spectrum of frequency and *in relation to others* (e.g., relying exclusively on one or two strategies versus using all strategies to a similar extent across stressors), which differs from the repertoire approach that more crudely aggregates across independent strategies considered in a binary way (more used/less or not used). Thus, the Between-Strategy Index captures attempts to find the best strategy or favor certain strategies for a given situation in a more nuanced way than the repertoire approach.

Although the current dispositional Between-Strategy Index is derived from a one-time assessment of how individuals *typically* cope with stressors rather than repeated assessments across *specific* situations, prior COPE research found moderate-to-high concordance between dispositional and situational versions of the inventory [2, 11]. Moreover, although a one-time assessment does not permit examination of dynamic changes in coping at a more granular level (e.g., within-strategy variability across days/stressors [10]), such single dispositional coping assessment can be informative when investigating long-term health outcomes [12] and has been validated and related to longevity outcomes in similar prior epidemiological studies, including [3]. The dispositional Between-Strategy Index was calculated as:

${SD}_{(between)i}=\sqrt{\frac{1}{L-1}\times\sum_{s=1}^{L} \left( x_{si}-M_{\left( between \right)i} \right)^{2}}$

where M_(between)i_ corresponds to the mean frequency score across coping subscales and $x_{si}$ corresponds to the subscale score denoting the degree of use of strategy *s* by individual *i* for the total number of strategies *L*.

**Supplemental Table 1.** Comparison of women who did (eligible) versus did not (non-eligible) complete the coping items on demographic, medical, and behavioral characteristics at baseline.

|  |  |  |  |
| --- | --- | --- | --- |
|  | |  |  |
|  | | Eligible (n=64,857) | Non-eligible (n=3,508) |
| *Demographic characteristics* | |  |  |
| Age, M(SD) | | 46.3 (4.7) | 46.9 (4.6) |
| White, % | | 96.8 | 96.3 |
| Married, % | | 79.7 | 79.6 |
| Census tract income in thousands of dollars, M(SD) | | 66 (24) | 67 (24) |
|  | |  |  |
| *Health-related factors* | |  |  |
| Body mass index <25 kg/m^2^, % | | 48.6 | 50.2 |
| Physical exam in last two years, % | | 88.1 | 86.9 |
|  | |  |  |
| *Health behaviors* | |  |  |
| Current non-smoker | | 91.6 | 92.2 |
| ≥150 min/week of moderate-to-vigorous physical activity, % | | 44.1 | 43.5 |
| Favorable AHEI diet score (in top 40 percent), % | | 40.3 | 45.7 |
| Alcohol intake 0 drink/day, % | | 39.7 | 38.8 |
| Sleep 7-8 h/night, % | | 65.8 | 65.6 |
| *Notes.* Values are means (SD) for continuous variables and percentages for categorical variables. Values of polytomous variables may not sum to 100% due to rounding. AHEI=Alternative Healthy Eating Index.  Missing data in the eligible sample: married=2425, body mass index=4879, physical exam=3409, smoking=3, physical activity=3322, diet=3153, alcohol=3153, sleep=2478; Missing data in the non-eligible sample: married=178, body mass index=327, physical exam=230, smoking=3, physical activity=235, diet=235, alcohol=235, sleep=179. | | | |
|  | | | |

**Supplemental Table 2.** Comparison of women who completed all versus some follow-up lifestyle assessments (score including the sleep component) on demographic, medical, behavioral, and psychological characteristics at baseline.

|  |  |  |  |
| --- | --- | --- | --- |
|  | | All (n=45,334) | Some (n=733) |
| *Demographic characteristics* | |  |  |
| Age, M(SD) | | 46.2 (4.7) | 47.0 (4.5) |
| White, % | | 97.3 | 93.9 |
| Married, % | | 80.8 | 81.3 |
| Census tract income in thousands of dollars, M(SD) | | 66 (24) | 65 (23) |
|  | |  |  |
| *Health-related factors* | |  |  |
| Body mass index ≤25 kg/m^2^, % | | 50.6 | 42.0 |
| Physical exam in last two years, % | | 87.5 | 86.9 |
|  | |  |  |
| *Health behaviors* | |  |  |
| Current non-smoker | | 92.4 | 90.0 |
| ≥150 min/week of moderate-to-vigorous physical activity, % | | 45.2 | 48.7 |
| Favorable AHEI diet score (in top 40 percent), % | | 40.2 | 39.3 |
| Alcohol intake 0 drink/day, % | | 38.7 | 44.1 |
| Sleep 7-8 h/night, % | | 67.2 | 63.8 |
|  | |  |  |
| *Coping strategies* | |  |  |
| Active Coping | | 5.1 (1.1) | 5.1 (1.1) |
| Acceptance | | 4.9 (1.0) | 4.9 (1.1) |
| Religion | | 3.9 (1.9) | 4.0 (2.0) |
| Using Emotional Support | | 4.5 (1.4) | 4.5 (1.4) |
| Focusing on & Venting of Emotion | | 3.4 (1.4) | 3.3 (1.4) |
| Denial | | 0.4 (0.7) | 0.5 (0.8) |
| Behavioral Disengagement | | 0.5 (0.8) | 0.5 (0.9) |
| Self-Blame | | 2.4 (1.4) | 2.3 (1.5) |
| *Notes.* Values are means (SD) for continuous variables and percentages for categorical variables. Values of polytomous variables may not sum to 100% due to rounding. AHEI=Alternative Healthy Eating Index. | | | |
|  | | | |

**Supplemental Table 3.** Descriptive statistics and correlation matrix of the coping individual strategies and variability levels.

|  |  | 1. | 2. | | 3. | | 4. | | 5. | | 6. | | 7. | | 8. | | 9. | | 10. | | 11. |
| --- | --- | --- | --- | --- | --- | --- | --- | --- | --- | --- | --- | --- | --- | --- | --- | --- | --- | --- | --- | --- | --- |
| *Individual coping strategies  (per 1-unit)* | *Mean (SD)* |  |  | |  | |  | |  | |  | |  | |  | |  | |  | |  |
| 1. Active Coping | 5.1 (1.1) | 1 | 0.28 | | 0.15 | | 0.30 | | 0.14 | | -0.12 | | -0.35 | | -0.19 | | -0.53 | | 0.08 | | 0.45 |
| 2. Acceptance | 4.9 (1.0) |  | 1 | | 0.19 | | 0.16 | | 0.09 | | -0.16 | | -0.15 | | -0.07 | | -0.44 | | 0.02 | | 0.42 |
| 3. Religion | 3.9 (1.9) |  |  | | 1 | | 0.22 | | 0.03 | | -0.04 | | -0.08 | | -0.04 | | -0.28 | | 0.00 | | 0.28 |
| 4. Use of Emotional Support | 4.5 (1.4) |  |  | |  | | 1 | | 0.25 | | -0.02 | | -0.19 | | -0.13 | | -0.38 | | -0.02 | | 0.40 |
| 5. Focus on & Venting of Emotions | 3.4 (1.4) |  |  | |  | |  | | 1 | | 0.01 | | 0.01 | | 0.13 | | -0.15 | | -0.01 | | 0.16 |
| 6. Denial | 0.4 (0.7) |  |  | |  | |  | |  | | 1 | | 0.27 | | 0.18 | | 0.34 | | -0.05 | | -0.29 |
| 7. Behavioral Disengagement | 0.5 (0.8) |  |  | |  | |  | |  | |  | | 1 | | 0.33 | | 0.45 | | -0.07 | | -0.38 |
| 8. Self-Blame | 2.4 (1.4) |  |  | |  | |  | |  | |  | |  | | 1 | | 0.21 | | 0.04 | | -0.25 |
| *Variability in coping strategies used  (dichotomized)* | | | |  | |  | |  | |  | |  | |  | |  | |  | |  |  |
| 9. Lower |  |  |  | |  | |  | |  | |  | |  | |  | | 1 | | -0.50 | | -0.50 |
| 10. Moderate |  |  |  | |  | |  | |  | |  | |  | |  | |  | | 1 | | -0.50 |
| 11. Greater |  |  |  | |  | |  | |  | |  | |  | |  | |  | |  | | 1 |

*Notes.* All coefficients are statistically significant at *p*≤0.05, except for the correlation of -.01 between Religion and Moderate Variability. SD=standard deviation. Individual coping strategies and mean level across strategies scores range from 0 to 6. Correlations using continuous data used Pearson correlations, whereas correlations using categorical data used Spearman correlations.

**Supplemental Table 4.** Relative risk and 95% confidence interval of sustained healthy smoking behavior associated with the adoption of coping individual strategies and variability levels

|  | Model 1: Age  RR (95% CI) | Model 2: Demographics  RR (95% CI) | Model 3: Demographics  + health-related factors  RR (95% CI) |
| --- | --- | --- | --- |
| *Individual coping strategies (per 1-SD increase)* | |  |  |
| Active Coping | 1.00 (1.00, 1.01)*** | 1.00 (1.00, 1.01)*** | 1.00 (1.00, 1.01)*** |
| Acceptance | 1.00 (1.00, 1.00) | 1.00 (1.00, 1.00) | 1.00 (1.00, 1.00) |
| Religion | 1.01 (1.01, 1.01)*** | 1.01 (1.01, 1.01)*** | 1.01 (1.01, 1.02)*** |
| Use of Emotional Support | 1.01 (1.00, 1.01)*** | 1.01 (1.00, 1.01)*** | 1.00 (1.00, 1.01)*** |
| Focus on & Venting of Emotions | 1.00 (1.00, 1.00)* | 1.00 (1.00, 1.00)** | 1.00 (1.00, 1.00)** |
| Denial | 0.99 (0.99, 0.99)*** | 0.99 (0.99, 0.99)*** | 0.99 (0.99, 0.99)*** |
| Behavioral Disengagement | 0.99 (0.99, 0.99)*** | 0.99 (0.99, 0.99)*** | 0.99 (0.99, 0.99)*** |
| Self-Blame | 1.00 (0.99, 1.00)*** | 1.00 (0.99, 1.00)*** | 1.00 (0.99, 1.00)*** |
| *Variability in coping strategies used* |  |  |  |
| Moderate versus lower variability | 1.01 (1.01, 1.02)*** | 1.01 (1.01, 1.02)*** | 1.01 (1.01, 1.02)*** |
| Greater versus lower variability | 1.02 (1.01, 1.02)*** | 1.02 (1.01, 1.02)*** | 1.02 (1.01, 1.02)*** |
| Greater versus moderate variability | 1.01 (1.00, 1.01)** | 1.01 (1.00, 1.01)** | 1.01 (1.00, 1.01)** |

*Notes.* N=46,067, n_events_=44,185 (where events=individuals who had a healthy smoking behavior at ≥2 time points over the follow-up period; number of events per variability levels: lower=14,398, moderate=14,915, greater=14,872).

The first four individual coping strategies are typically considered more adaptive whereas the last four strategies are typically deemed less adaptive. Although individual coping strategies and coping variability levels are presented in the same table, they represent distinct analyses.

**p*≤0.05; ***p*≤0.01; ****p*≤0.001. CI=confidence interval, RR=relative risk, SD=standard deviation. Significant CI including the 1.00 value are due to rounding.

Model 1 adjusted for age. Model 2 adjusted for age, race, census tract income, and marital status. Model 3 (core) adjusted for Model 2 as well as body mass index and physical exam within the last 2 years. All coping variability models further adjusted for mean of all individual coping strategies.

**Supplemental Table 5.** Relative risk and 95% confidence interval of sustained healthy diet associated with the adoption of coping individual strategies and variability levels

|  | Model 1: Age  RR (95% CI) | Model 2: Demographics  RR (95% CI) | Model 3: Demographics  + health-related factors  RR (95% CI) |
| --- | --- | --- | --- |
| *Individual coping strategies (per 1-SD increase)* | |  |  |
| Active Coping | 1.09 (1.08, 1.11)*** | 1.09 (1.08, 1.10)*** | 1.08 (1.07, 1.09)*** |
| Acceptance | 1.02 (1.01, 1.03)** | 1.02 (1.01, 1.03)*** | 1.02 (1.01, 1.03)*** |
| Religion | 1.00 (0.99, 1.01) | 1.02 (1.01, 1.03)*** | 1.02 (1.01, 1.03)*** |
| Use of Emotional Support | 1.07 (1.06, 1.08)*** | 1.07 (1.06, 1.08)*** | 1.06 (1.05, 1.07)*** |
| Focus on & Venting of Emotions | 1.04 (1.03, 1.05)*** | 1.03 (1.02, 1.04)*** | 1.03 (1.02, 1.04)*** |
| Denial | 0.96 (0.95, 0.97)*** | 0.96 (0.95, 0.97)*** | 0.96 (0.95, 0.97)*** |
| Behavioral Disengagement | 0.95 (0.94, 0.96)*** | 0.95 (0.94, 0.96)*** | 0.95 (0.94, 0.96)*** |
| Self-Blame | 0.95 (0.94, 0.96)*** | 0.96 (0.95, 0.96)*** | 0.96 (0.95, 0.97)*** |
| *Variability in coping strategies used* |  |  |  |
| Moderate versus lower variability | 1.12 (1.10, 1.15)*** | 1.12 (1.10, 1.15)*** | 1.11 (1.09, 1.14)*** |
| Greater versus lower variability | 1.19 (1.16, 1.22)*** | 1.19 (1.16, 1.22)*** | 1.17 (1.15, 1.20)*** |
| Greater versus moderate variability | 1.06 (1.04, 1.08)*** | 1.06 (1.04, 1.08)*** | 1.05 (1.03, 1.08)*** |

*Notes.* N=46,067, n_events_=22,357 (where events=individuals who had a healthy diet level at ≥2 time points over the follow-up period; number of events per variability levels: lower=6,649, moderate=7,648, greater=8,060).

The first four individual coping strategies are typically considered more adaptive whereas the last four strategies are typically deemed less adaptive. Although individual coping strategies and coping variability levels are presented in the same table, they represent distinct analyses.

**p*≤0.05; ***p*≤0.01; ****p*≤0.001. CI=confidence interval, RR=relative risk, SD=standard deviation. Significant CI including the 1.00 value are due to rounding.

Model 1 adjusted for age. Model 2 adjusted for age, race, census tract income, and marital status. Model 3 (core) adjusted for Model 2 as well as body mass index and physical exam within the last 2 years. All coping variability models further adjusted for mean of all individual coping strategies.

**Supplemental Table 6.** Relative risk and 95% confidence interval of sustained healthy physical activity associated with the adoption of coping individual strategies and variability levels

|  | Model 1: Age  RR (95% CI) | Model 2: Demographics  RR (95% CI) | Model 3: Demographics  + health-related factors  RR (95% CI) |
| --- | --- | --- | --- |
| *Individual coping strategies (per 1-SD increase)* | |  |  |
| Active Coping | 1.11 (1.10, 1.12)*** | 1.10 (1.09, 1.11)*** | 1.09 (1.08, 1.10)*** |
| Acceptance | 1.03 (1.02, 1.03)*** | 1.03 (1.02, 1.04)*** | 1.02 (1.02, 1.03)*** |
| Religion | 1.01 (1.00, 1.02) | 1.02 (1.01, 1.03)*** | 1.02 (1.01, 1.02)*** |
| Use of Emotional Support | 1.06 (1.05, 1.07)*** | 1.05 (1.04, 1.06)*** | 1.05 (1.04, 1.06)*** |
| Focus on & Venting of Emotions | 1.02 (1.01, 1.03)*** | 1.02 (1.01, 1.03)*** | 1.02 (1.01, 1.03)*** |
| Denial | 0.97 (0.96, 0.98)*** | 0.97 (0.96, 0.98)*** | 0.98 (0.97, 0.98)*** |
| Behavioral Disengagement | 0.92 (0.91, 0.93)*** | 0.92 (0.91, 0.93)*** | 0.93 (0.92, 0.94)*** |
| Self-Blame | 0.95 (0.95, 0.96)*** | 0.96 (0.95, 0.96)*** | 0.96 (0.96, 0.97)*** |
| *Variability in coping strategies used* |  |  |  |
| Moderate versus lower variability | 1.13 (1.11, 1.15)*** | 1.13 (1.11, 1.15)*** | 1.11 (1.09, 1.13)*** |
| Greater versus lower variability | 1.19 (1.17, 1.21)*** | 1.19 (1.16, 1.21)*** | 1.16 (1.14, 1.18)*** |
| Greater versus moderate variability | 1.05 (1.04, 1.07)*** | 1.05 (1.03, 1.07)*** | 1.04 (1.03, 1.06)*** |

*Notes.* N=46,067, n_events_=26,687 (where events=individuals who had a healthy physical activity level at ≥2 time points over the follow-up period; number of events per variability levels: lower=7,948, moderate=9,168, greater=9,571).

The first four individual coping strategies are typically considered more adaptive whereas the last four strategies are typically deemed less adaptive. Although individual coping strategies and coping variability levels are presented in the same table, they represent distinct analyses.

**p*≤0.05; ***p*≤0.01; ****p*≤0.001. CI=confidence interval, RR=relative risk, SD=standard deviation. Significant CI including the 1.00 value are due to rounding.

Model 1 adjusted for age. Model 2 adjusted for age, race, census tract income, and marital status. Model 3 (core) adjusted for Model 2 as well as body mass index and physical exam within the last 2 years. All coping variability models further adjusted for mean of all individual coping strategies.

**Supplemental Table 7.** Relative risk and 95% confidence interval of sustained healthy alcohol consumption associated with the adoption of coping individual strategies and variability levels.

|  | Model 1: Age  RR (95% CI) | Model 2: Demographics  RR (95% CI) | Model 3: Demographics  + health related-factors  RR (95% CI) |
| --- | --- | --- | --- |
| *Individual coping strategies (per 1-SD increase)* | |  |  |
| Active Coping | 0.96 (0.95, 0.97)*** | 0.97 (0.96, 0.98)*** | 0.97 (0.96, 0.98)*** |
| Acceptance | 1.04 (1.03, 1.05)*** | 1.04 (1.03, 1.05)*** | 1.04 (1.03, 1.06)*** |
| Religion | 1.20 (1.19, 1.22)*** | 1.18 (1.16, 1.19)*** | 1.18 (1.16, 1.19)*** |
| Use of Emotional Support | 0.96 (0.95, 0.97)*** | 0.97 (0.96, 0.98)*** | 0.97 (0.96, 0.98)*** |
| Focus on & Venting of Emotions | 0.95 (0.94, 0.96)*** | 0.95 (0.94, 0.97)*** | 0.95 (0.94, 0.97)*** |
| Denial | 1.00 (0.99, 1.01) | 0.99 (0.98, 1.00) | 0.99 (0.98, 1.00) |
| Behavioral Disengagement | 1.04 (1.03, 1.05)*** | 1.03 (1.02, 1.04)*** | 1.03 (1.02, 1.04)*** |
| Self-Blame | 1.01 (1.00, 1.02) | 1.01 (1.00, 1.02) | 1.00 (0.99, 1.01) |
| *Variability in coping strategies used* |  |  |  |
| Moderate versus lower variability | 1.02 (0.99, 1.05) | 1.02 (0.99, 1.05) | 1.03 (1.00, 1.06)* |
| Greater versus lower variability | 1.09 (1.06, 1.12)*** | 1.10 (1.07, 1.13)*** | 1.12 (1.09, 1.15)*** |
| Greater versus moderate variability | 1.08 (1.05, 1.11)*** | 1.08 (1.05, 1.11)*** | 1.09 (1.06, 1.12)*** |

*Notes.* N=46,067, n_events_= 18,275 (where events=individuals who had a healthy alcohol consumption at ≥2 time points over the follow-up period; number of events per variability levels: lower=5,807, moderate= 6,036, greater=6,432).

The first four individual coping strategies are typically considered more adaptive whereas the last four strategies are typically deemed less adaptive. Although individual coping strategies and coping variability levels are presented in the same table, they represent distinct analyses.

**p*≤0.05; ***p*≤0.01; ****p*≤0.001. CI=confidence interval, RR=relative risk, SD=standard deviation. Significant CI including the 1.00 value are due to rounding.

Model 1 adjusted for age. Model 2 adjusted for age, race, census tract income, and marital status. Model 3 (core) adjusted for Model 2 as well as body mass index and physical exam within the last 2 years. All coping variability models further adjusted for mean of all individual coping strategies.

**Supplemental Table 8.** Relative risk and 95% confidence interval of sustained healthy sleep behavior^§^ associated with the adoption of coping individual strategies and variability levels

|  | Model 1: Age  RR (95% CI) | Model 2: Demographics  RR (95% CI) | Model 3: Demographics  + health-related factors  RR (95% CI) |
| --- | --- | --- | --- |
| *Individual coping strategies (per 1-SD increase)* | |  |  |
| Active Coping | 1.12 (1.10, 1.14)*** | 1.12 (1.10, 1.14)*** | 1.11 (1.09, 1.13)*** |
| Acceptance | 1.03 (1.01, 1.05)*** | 1.03 (1.02, 1.05)*** | 1.03 (1.01, 1.05)*** |
| Religion | 1.06 (1.04, 1.08)*** | 1.06 (1.04, 1.08)*** | 1.06 (1.04, 1.08)*** |
| Use of Emotional Support | 1.12 (1.10, 1.14)*** | 1.11 (1.10, 1.13)*** | 1.11 (1.09, 1.13)*** |
| Focus on & Venting of Emotions | 1.00 (0.98, 1.01) | 1.00 (0.98, 1.01) | 0.99 (0.98, 1.01) |
| Denial | 0.94 (0.93, 0.96)*** | 0.95 (0.93, 0.96)*** | 0.95 (0.93, 0.97)*** |
| Behavioral Disengagement | 0.87 (0.86, 0.89)*** | 0.88 (0.86, 0.89)*** | 0.88 (0.86, 0.90)*** |
| Self-Blame | 0.82 (0.81, 0.84)*** | 0.83 (0.81, 0.84)*** | 0.83 (0.81, 0.84)*** |
| *Variability in coping strategies used* |  |  |  |
| Moderate versus lower variability | 1.18 (1.13, 1.23)*** | 1.18 (1.13, 1.23)*** | 1.17 (1.12, 1.22)*** |
| Greater versus lower variability | 1.33 (1.28, 1.39)*** | 1.33 (1.28, 1.38)*** | 1.31 (1.26, 1.37)*** |
| Greater versus moderate variability | 1.13 (1.09, 1.17)*** | 1.13 (1.09, 1.17)*** | 1.12 (1.08, 1.17)*** |

*Notes.* N=46,059, n_events_=11,159 (where events=individuals who had a healthy sleep behavior at ≥2 time points over the follow-up period; number of events per variability levels: lower=3,138, moderate=3,782, greater=4,239).

The first four individual coping strategies are typically considered more adaptive whereas the last four strategies are typically deemed less adaptive. Although individual coping strategies and coping variability levels are presented in the same table, they represent distinct analyses.

**p*≤0.05; ***p*≤0.01; ****p*≤0.001. CI=confidence interval, RR=relative risk, SD=standard deviation. Significant CI including the 1.00 value are due to rounding.

Model 1 adjusted for age. Model 2 adjusted for age, race, census tract income, and marital status. Model 3 (core) adjusted for Model 2 as well as body mass index and physical exam within the last 2 years. All coping variability models further adjusted for mean of all individual coping strategies.^§^ Sleep was characterized with quality and/or duration data, whenever the information was available.

**References**

1. Eisinga, R., M. Grotenhuis, and B. Pelzer, *The reliability of a two-item scale: Pearson, Cronbach, or Spearman-Brown?* International Journal of Public Health, 2013. **58**(4): p. 637-42.

2. Carver, C.S., M.F. Scheier, and J.K. Weintraub, *Assessing coping strategies: A theoretically based approach.* Journal of Personality and Social Psychology, 1989. **56**(2): p. 267-83.

3. Trudel-Fitzgerald, C., et al., *Are coping strategies and variability in their use associated with lifespan?* Journal of Psychosomatic Research, 2022. **162**: p. 111035.

4. Kato, T., *Frequently used coping scales: A meta-analysis.* Stress & Health, 2015. **31**(4): p. 315-23.

5. Greenaway, K.H., et al., *Successful coping for psychological well-being*, in *Measures of personality and social psychological constructs*, G. Boyle, D.H. Saklofske, and G. Matthews, Editors. 2014, Elsevier: Oxford. p. 322-351.

6. Trudel-Fitzgerald, C., et al., *Coping and emotion regulation: A conceptual and measurement scoping review.* Canadian Psychology / Psychologie Canadienne, 2024. **65**(3): p. 149-162.

7. Carver, C.S. *Self-reported measures available: COPE*. 2019 August, 2020]; Available from: <https://local.psy.miami.edu/people/faculty/ccarver/availbale-self-report-instruments/cope/>.

8. Cheng, C., H.P. Lau, and M.P. Chan, *Coping flexibility and psychological adjustment to stressful life changes: A meta-analytic review.* Psychological Bulletin, 2014. **140**(6): p. 1582-607.

9. Bonanno, G.A. and C.L. Burton, *Regulatory flexibility: An individual differences perspective on coping and emotion regulation.* Perspectives on Psychological Sciences, 2013. **8**(6): p. 591-612.

10. Blanke, E.S., et al., *Mix it to fix it: Emotion regulation variability in daily life.* Emotion, 2020. **20**(3): p. 473-485.

11. Carver, C.S. and M.F. Scheier, *Situational coping and coping dispositions in a stressful transaction.* Journal of Personality and Social Psychology, 1994. **66**(1): p. 184-95.

12. Lazarus, R.S., *Theory-based stress measurement.* Psychological Inquiry, 1990. **1**: p. 3-13.
